# Systems Virology and Human Cytomegalovirus: Using High Throughput Approaches to Identify Novel Host-Virus Interactions During Lytic Infection

**DOI:** 10.3389/fcimb.2020.00280

**Published:** 2020-06-10

**Authors:** Chen-Hsuin Lee, Finn Grey

**Affiliations:** Division of Infection and Immunity, Roslin Institute, The University of Edinburgh, Edinburgh, United Kingdom

**Keywords:** human cytomegalovirus, high-throughput, HTS, RNAi, siRNA screen

## Abstract

Human Cytomegalovirus (HCMV) is a highly prevalent herpesvirus, persistently infecting between 30 and 100% of the population, depending on socio-economic status (Fields et al., 2013). HCMV remains an important clinical pathogen accounting for more than 60% of complications associated with solid organ transplant patients (Kotton, 2013; Kowalsky et al., 2013; Bruminhent and Razonable, 2014). It is also the leading cause of infectious congenital birth defects and has been linked to chronic inflammation and immune aging (Ballard et al., 1979; Griffith et al., 2016; Jergovic et al., 2019). There is currently no effective vaccine and HCMV antivirals have significant side effects. As current antivirals target viral genes, the virus can develop resistance, reducing drug efficacy. There is therefore an urgent need for new antiviral agents that are effective against HCMV, have better toxicity profiles and are less vulnerable to the emergence of resistant strains. Targeting of host factors that are critical to virus replication is a potential strategy for the development of novel antivirals that circumvent the development of viral resistance. Systematic high throughput approaches provide powerful methods for the identification of novel host-virus interactions. As well as contributing to our basic understanding of virus and cell biology, such studies provide potential targets for the development of novel antiviral agents. High-throughput studies, such as RNA sequencing, proteomics, and RNA interference screens, are useful tools to identify HCMV-induced global changes in host mRNA and protein expression levels and host factors important for virus replication. Here, we summarize new findings on HCMV lytic infection from high-throughput studies since 2014 and how screening approaches have evolved.

## Introduction

Human cytomegalovirus (HCMV) is a ubiquitous human pathogen that belongs to the β-herpesvirus subfamily. It has the largest DNA genome among the human herpesviruses with ~235 kb pairs, and encodes at least 173 annotated genes based on the low-passage strain Merlin, the current reference strain for wild-type HCMV (Dunn et al., 2003; Fields et al., 2007; Sijmons et al., 2014; Marti-Carreras and Maes, 2019). HCMV can infect a broad range of cell types, including endothelial and epithelial cells, fibroblast cells and cells derived from the myeloid lineages (Rice et al., 1984; Sinzger et al., 2008). It has a relatively long lytic replication cycle which can take up to 72 h to start releasing infectious virions into the extracellular compartment (Landolfo et al., 2003). A lifelong latent infection can be established in primitive bone-marrow-resident CD34+ hematopoietic stem cells and CD33+ myeloid progenitor cells which retain the latent viral genome as they differentiate into peripheral blood CD14+ monocytes and myeloid dendritic cells (mDCs) (Collins-McMillen et al., 2018; Arcangeletti et al., 2016; Stern et al., 2019). Reactivation of the latent viral genome occurs when latently infected cells reach terminal differentiation to mDCs and macrophages, accompanied by chromatin remodeling of the HCMV major immediate-early promoter (Reeves et al., 2005). Other factors such as allogeneic stimulation and pro-inflammatory cytokines such as interferon gamma (IFN-γ) have also been implicated in driving the maturation of myeloid cells and reactivation of latently infected cells (Stern et al., 2019).

Viruses are obligate intracellular pathogens which rely on host cell machinery to infiltrate, replicate in, and egress target cells. HCMV has complex interactions with various host factors, and host-virus interactions vary among permissive cell types as well as between lytic and latent stages of virus infection. Characterizing changes in host RNA expression and proteomes upon infection can help dissect these complex host-virus interactions, as the virus is likely to induce host factors required for efficient virus replication and to suppress factors that would otherwise inhibit virus replication. Studying how the virus interacts with the host can contribute to a better understanding of virus biology as well as functions of host factors. Furthermore, host factors that are identified as critical for virus replication can be targeted by small molecule inhibitors, thereby leading to potentially novel HCMV antiviral therapies.

High-throughput studies are powerful tools to systematically study host-virus interactions. There are many strategies to dissect the complex interactions between a virus and host cells. RNA sequencing and mass spectrometry allow the temporal and spatial expression of viral and host factors to be monitored during the course of infection, subsequently discovering the mechanism of viral manipulation of host machinery and the host factors important for virus replication (Weekes et al., 2014; Jean Beltran et al., 2016). In addition, the expression of host genes can be systematically silenced through the RNA-induced silencing complex (RISC) or disrupted via clustered regularly interspaced short palindromic repeats (CRISPR) technology (Polachek et al., 2016; McCormick et al., 2018). The resulting effects of individual gene depletion or disruption on virus replication can be examined using a reporter system such as luminescence, enhanced green fluorescence protein (eGFP), or immunofluorescence staining for viral proteins. Finally, instead of silencing gene expression, host genes, such as interferon stimulated genes (ISGs) can be overexpressed using arrayed lentivirus libraries and host restriction factors against the virus can be identified (Schoggins et al., 2011).

Previously, a thorough review was published in 2014, discussing how HCMV manipulates host metabolism and gene expression using RNA sequencing (RNA-seq), proteomics and high-throughput RNAi screens (Cohen and Stern-Ginossar, 2014). However, studies using high throughput or systematic approaches have made major contributions to our understanding of HCMV biology, both in the lytic and latent aspects, in the last 5 years. Therefore, in this review, we summarize high-throughput studies in HCMV, focusing on lytic infection, from 2014 to current. The review will be in two major parts: (i) how the virus changes cellular RNA and protein expression, and (ii) how changes to host cells impact virus replication.

## Global Analysis of HCMV-Induced Changes in Host Gene Expression by RNA-SEQ and Proteomics

Upon infection, HCMV induces global changes in host protein expression to overcome host innate immune defenses and divert normal cellular processes away from the host to the virus to facilitate efficient virus replication. These changes may be observed at RNA levels where viral proteins directly interact with the host DNA or chromatin or indirectly to increase or decrease specific host gene expression, thereby creating an environment conducive to efficient replication and spread. However, mRNA transcripts are subject to post-transcriptional regulation and degradation, and the levels of transcripts and proteins do not always correlate. Therefore, analyzing changes in protein levels upon infection can demonstrate whether alterations in mRNA level correspond to changes in protein levels and whether the changes in protein expression have roles and functions in HCMV replication.

### RNA-seq Studies in HCMV

RNA-sequencing (RNA-seq) has revolutionized our ability to monitor gene expression levels on a genome wide basis. Techniques such as purification of poly-A transcripts or depletion of ribosomal RNA (rRNA) have improved the sensitivity and accuracy of RNA-seq (Zhao et al., 2018). In addition to using RNA-seq to quantify RNA levels, novel strategies have been developed to investigate post-transcriptional events including alternative splicing (AS) and alternative cleavage and polyadenylation (APA), as these events determine the coding potentials of mRNA transcripts and regulate gene expression. AS is a tightly regulated process that generates multiple mRNA isoforms by using different combinations of splice sites and exons. Several classes of splicing patterns include exon skipping, alternative 5′ splice site selection, and retention of introns (Movassat et al., 2016). Meanwhile, APA involves the use of alternative poly(A) sites located in internal introns or exons to form multiple mRNA isoforms.

Polyadenylation is a two-step process involving specific endonucleolytic cleavage and polymerisation of the adenosine tail of which length is host-specific and is often about 200 residues in higher eukaryotes (Lutz and Moreira, 2011). Poly(A) tails are found on the 3′ end of nearly every eukaryotic mRNA transcript and a particular poly(A) site is chosen depending on the strength of the poly(A) signal and surrounding *cis*-elements (Millevoi and Vagner, 2010; Fu and Ares, 2014). APA can also occur in the 3′ untranslated regions (3′UTR) resulting in transcription with different 3′UTR lengths, which has been suggested to affect the stability, localization, transport and translation of the mRNA (Movassat et al., 2016).

AS has been identified in around 95% of human genes whilst APA in about 70% of human genes (Pan et al., 2008; Derti et al., 2012). AS and APA can affect many cellular processes including RNA stability, translation, miRNA targeting, development, and disease (Lutz and Moreira, 2011). RNA-seq was used to study how AS and APA alters the host transcriptomes at the post-transcription level following infection with HCMV. Batra et al. (2016) assessed mRNA expression patterns in three HCMV-permissive cell types using splicing-sensitive Affymetrix microarrays and poly-A selected RNA-seq. RNAs were extracted from primary human foreskin fibroblast cells (HFFs), human aortic endothelial cells (ECs), and human embryonic stem cell derived neural progenitor cells (NPCs) at 48 and 96 h post-infection (HPI) with HCMV strain TB40/E. Changes in APA and AS resulted in over 2,000 alterations in host transcripts following infection with HCMV. Using Mixture of isoforms (MISO) algorithm, it was found that the majority of APA events resulted in shortened 3′UTRs post-infection in all three cell types tested, with 3′UTRs shorten by up to 5 kilobases (kb) at 96 HPI. To evaluate the regulation of APA at the 3′UTR, RNA-seq analysis revealed a drastic upregulation of cytoplasmic polyadenylation element binding protein 1 (CPEB1) mRNA levels, which corresponded to increased protein expression. Depletion of CPEB1 by small interfering RNA (siRNA) during infection partially or completely reversed 41% of APA changes in infected HFFs, whilst overexpression of CPEB1 resulted in a significant shift toward utilization of proximal poly(A) sites in genes found to be affected during infection. Furthermore, depletion of CPEB1 also shortened poly(A) tails in HCMV genes, such as UL18 and UL99, and decreased productive HCMV infection in HFFs, although the magnitude of decrease was more substantial at 96 HPI (around 10-fold) and the titer recovered partially by 144 HPI. This study demonstrates the power of systematic approaches for understanding how viruses can manipulate gene expression levels on a global scale.

### Proteomics Studies in HCMV

mRNA levels don't always correspond to protein levels due to various factors, including post-transcriptional modification, mRNA stability, and regulation by miRNAs (Valencia-Sanchez et al., 2006; Nachtergaele and He, 2017; Nouaille et al., 2017; Mauger et al., 2019). Therefore, measuring protein levels during infection can provide a more direct functional relationship between the virus and the host (Koussounadis et al., 2015; Liu et al., 2016). A seminal and quantitative proteomic study on the precise temporal expression of plasma membrane proteins and intracellular proteins was conducted by Weekes et al. (2014). Tandem mass tags (TMT) were used to quantify 1,184 cell-surface proteins and 7,491 cellular proteins from up to 10 time points in HFFs. Several cell-surface and cellular proteins in the interferon (IFN) signaling pathway were found to be downregulated by HCMV strain Merlin, including Jak1, STAT2, and IRF9. More recently, a similar strategy was adapted to focus on host proteins with innate immune function that are actively degraded by the proteasome or lysosome during early-stage HCMV infection (Nightingale et al., 2018). Using the proteasome inhibitor MG132, or leupeptin, an inhibitor against lysosome function, HFFs were infected with Merlin strain and analyzed at 12, 18, and 24 HPI by MS3 mass spectrometry. Eight thousand six hundred and seventy-eight proteins were assessed between infected cells treated with or without the proteasomal or lysosomal inhibitor. Overall, 131 proteins, including E3 ubiquitin ligase ANAPC1 and HCMV restriction factor Sp100, were rescued by application of MG132 within 24 HPI, with 46 proteins rescued at 12 HPI. Meanwhile, 28 proteins, including E3 ligase NEDD4, were rescued by application of leupeptin. Fifty percentage of proteins rescued by leupeptin were also rescued by MG132. To address protein stability and turnover during HCMV infection, an unbiased pulsed stable isotope labeling with amino acids in cell culture (pSILAC) with the chemical tag TMT was used. In comparison to conventional pSILAC approach, the combination of pSILAC with TMT allows better time efficiency in mass spectrometry and higher accuracy of protein measurement of each protein at every time point. The multiplexed pSILAC/TMT approach involves labeling cells with “medium” lysine (^2^H_4_ L-lysine; Lys4), providing cells with “heavy” lysine (^13^C_6_^15^N_2_ L-lysine; Lys8) at the time of infection to identify newly synthesized peptides, and labeling peptides with TMT for mass spectrometry. pSILAC results showed that 24 of 46 proteins rescued at 12 HPI with MG132 exhibited increased degradation in HCMV-infected cells compared to mock infection, whilst 12 proteins did not exhibit instability or degradation. Some of these proteins had extremely high turnover rate, making it difficult to observe a difference between mock and HCMV infection (e.g., TXNIP), whereas some of these proteins were also rescued by the application of MG132 during mock infection (e.g., PHLDA1). In summary, proteins that were rescued by MG132 but did not exhibit instability by pSILAC may still be degraded, and post-transcriptional and post-translational regulations may have worked in combination to regulate the gene expression. In addition, RNA-seq analysis was performed to identify proteins for which expression was determined primarily by post-transcriptional regulation rather than mRNA transcription. Several candidates were shown to have upregulated transcription but downregulation of protein expression, including the HCMV restriction factor Sp100, an E3 ubiquitin ligase ANAPC1 and the helicase-like transcription factor HLTF. The authors further characterized HLTF, which was not previously reported to be targeted for proteasomal degradation by HCMV, and found that UL145 is necessary and sufficient to degrade HLTF. UL145 was also found to recruit the Cullin 4 (CUL4) E3 ligase complex to target HLTF to proteasomal degradation. Finally, HLTF was shown to act as a restriction factor similar to Sp100, at an early stage of virus replication.

Another proteomic study conducted by Jean Beltran et al. (2016) investigated both temporal and spatial changes in cellular protein expression during HCMV infection using subcellular fractionation and TMT-based mass spectrometry. The HCMV infection cycle was separated into five time points (24, 48, 72, 96, and 120 HPI) and six spatial fractions by density gradient ultracentrifugation. The study revealed that nearly 4,000 host proteins in HFFs had undergone subcellular reorganization during HCMV infection and the lysosomes were remodeled into two distinct subpopulations during infection. The authors followed up on one of the hits, myosin XVIIIA (MYO18A), and showed that MYO18A translocated from the plasma membrane to lysosomes during infection. Knockdown of MYO18A by siRNA also showed a modest reduction in virus replication, measured by IE1 positive infectious units. MYO18A is involved in actin retrograde flow at the cell lamella and vesicle trafficking. A model was thus proposed suggesting MYO18A is translocated from the plasma membrane to the virus assembly compartment to tether viral loaded vesicles and motor proteins. The same group further investigated the global changes in peroxisome biogenesis during HCMV infection using targeted quantitative proteomics (Jean Beltran et al., 2018). Sixty peroxisomal proteins that were found suitable for quantification by mass spectrometry in HFFs were analyzed at 0, 6, 24, 48, 72, and 120 HPI with HCMV strain AD169 using high-resolution parallel reaction monitoring assay for high resolution and high mass accuracy. Overall, the abundance of most peroxisomal proteins (52 of 60) increased from 24 HPI. The authors subsequently showed that the induction of peroxisomal protein expression does not require the expression of late genes and peroxisome biogenesis are required for efficient HCMV replication.

In addition to studies that explore the global changes of proteomes, a study by Arend et al. focused on the changes in activity and expression level of host kinases using a multiplexed kinase inhibitor bead-mass spectrometry (MIB-MS) (Arend et al., 2017). Using beads that contain immobilized pan kinase inhibitors for kinase capture, the study identified temporal changes in the complement of host protein kinases (i.e., the kinome) during HCMV infection. The laboratory adapted strain AD169 and the clinical relevant strain TB40/E were used to infect MRC-5 fibroblast cells in order to compare the changes in kinome induced by two different strains. Interestingly, both AD169 and TB40/E modify host kinome in a similar manner. The results showed that 53 kinases including cyclin-dependent kinases (e.g., CDK1, CDK7) and lipid kinases (e.g., PIK3C3, PI4K2B) had increased expression during infection. Meanwhile, 51 kinases, such as platelet-derived growth factor receptor alpha and beta (PDGFRα and PDGFRβ) and transforming growth factor beta receptor 1 and 2 (TGFBR1 and TGFBR2), had reduced expressing following infection. The study confirmed the importance of previously reported AMPK, mTOR and ERK/MAPK signaling pathways during HCMV infection, as well as identifying ephrin (Eph) receptor kinases as novel pathways affected by HCMV. Based on the MIB-MS kinome profiling data, the authors tested 13 kinase specific inhibitors that are currently in clinical use or trials for their potential to inhibit HCMV infection. An AD169 virus expressing pp28 protein fused to the GFP on its C-terminus (pp28-GFP) was used to monitor the level of virus infection following drug treatment. It was found that four inhibitors significantly decreased pp28-GFP expression. These are OTSSP167 [targeting maternal embryonic leucine zipper kinase (MELK)], dinaciclib (targeting CDK1, 2, 5, and 9), OTX015 (targeting bromodomain-containing proteins BRD2, 3, and 4), and LY2603618 [targeting checkpoint kinase 1 (CHEK1)]. Detailed characterization was performed on OTSSP167 and virus infection was shown to decrease by 1 log following drug treatment. This study demonstrates the potential of identifying antiviral targets using kinome profiling.

## Systematic Identification of Novel Host-Virus Interactions Using RNA Interference and CRISPR/CAS9

Global analysis of RNA and protein levels during HCMV infection can help understand how the virus manipulates cellular gene expression as well as how cells respond to infection. However, certain host factors may not have differential expression before and after infection, but their functions are important for efficient virus replication. With the development of RNA interference (RNAi) using small interfering RNA (siRNA) and short hairpin RNA (shRNA), expression of host factors can be systematically silenced and the effect of individual gene depletion on virus replication can be investigated. In addition, the recently developed and advanced CRISPR genome editing technology allows mutations to be introduced to target genes and disrupt protein expression of host factors that are important for virus replication.

siRNA screens are usually performed in arrayed fashion where each well in a microplate has a unique RNAi reagent targeting one gene. On the other hand, shRNA screens are often performed as pooled screens where all RNAi reagents are pooled together and introduced randomly to cells, where shRNAs can be detected by next generation sequencing (NGS) or barcode microarray following phenotypic selection. High-throughput RNAi screens have contributed greatly to our understanding of basic cell biology as well as complex host-virus interactions which can lead to development of novel therapeutics.

### siRNA Studies in HCMV

siRNAs are regulatory RNA duplexes, usually 21–23 nucleotides long, with 2 nucleotide overhangs on the 3′ ends. They bind to complementary mRNA transcripts, causing degradation (Rao et al., 2009). siRNAs usually have one mRNA target, and the guide strand of an siRNA is incorporated into the RNA-induced silencing complex (RISC) which uses the guide sequence to recognize a target transcript for degradation (Lam et al., 2015). Because of its simplicity and specificity, siRNA has been adapted in high-throughput screens to perform systematic loss-of-function studies in order to identify genes important for normal cellular functions or virus replication.

Important aspects to consider when attempting high throughput virus siRNA screens is whether the chosen readout will generate a broad dynamic range and accurately reflect virus replication and, ultimately, virus production. The dynamic range of a screen describes the signal to noise ratio. If the chosen readout for virus replication generates a maximal signal of 2-fold over background, the sensitivity of the screen will be poor as the largest phenotype possible would be a 2-fold reduction. In contrast, a screen that generates a 100-fold dynamic range provides far greater scope for defining phenotypic effects and increases the sensitivity of the screen. In addition, if the readout does not accurately reflect virus replication and, arguably the more critical measurement, virus production, the false discovery rate will be substantially higher. Conventional siRNA screens often use reporter constructs incorporated into the viral genome to generate a signal that reflects virus replication. While this approach allows for rapid, efficient and real-time measurement of signal, any effects on virus replication are biased toward early events such as entry and genome replication and may be less effective at measuring late events such as assembly and egress. Previously we showed that measuring viral replication based on expression of fluorescence cassette incorporated into the virus genome alone, was a poor predictor of effects on late aspects of virus replication (McCormick et al., 2018). Furthermore, basing the readout directly on a fluorescent reporter can result in the identification of genes that have a direct effect on the fluorescent signal rather than through inhibition or promotion of virus replication.

Another aspect to consider is the potential for off-target effects, where the observed phenotype is caused by inhibition of a gene other than the intended target. The use of pooled siRNAs has reduced problems of off-target effects. By using three to four independent siRNAs that target different regions of a transcript, concentrations of each siRNA can be reduced, thereby diminishing the risk of off target effects, while generating synergistic RNAi activity, improving efficiency and specificity of knockdown. However, careful validation is still required for any potential hit and pooled siRNAs can be deconvoluted during hit validation and individual siRNAs examined to confirm the specificity of knockdown.

In this section we describe a number of HCMV siRNA screens that have used alternative approaches to measure virus replication, enabling improved measurement of early and late aspects of HCMV virus replication.

Polachek et al. (2016) performed an siRNA screen to determine the role of cellular kinases and phosphatases on HMCV replication. In this study, 789 human kinases and phosphatases were targeted in HFFs using siRNA pools and the HCMV laboratory strain AD169. The immunofluorescence staining of viral antigen pp28 (UL99-encoded) at 72 HPI was employed as an indication of the level of viral replication. A high-throughput high-content confocal microscopy with an ImageXpress Micro microscope was used to quantify the level of pp28 expression, allowing image capturing in microplate systems. As pp28 is a late gene, visualization of this protein would more accurately reflect all stages of virus replication. The results showed that 25 siRNAs had enhancing effects on HCMV replication with *Z*-scores above 1 whilst 30 siRNAs had inhibitory effects with *Z*-scores below −1. *Z*-score describes how many standard deviations a given measurement lies above or below a population mean (Curtis et al., 2016). Only 4 siRNA pools targeting phosphatidylinositol 3-kinase class II alpha (PI3K-C2A), cluster of differentiation 4 (CD4), exosome component 10 (EXOSC10) and WNK lysine deficient protein kinase 4 (WNK4) were found to have strong inhibitory effects (*Z*-scores below −2) on HCMV replication. However, CD4 and WNK4 were not found to be expressed in HFFs infected with HCMV; thus, they were excluded from further analysis. PI3K-C2A was further characterized since it had not been previously linked to HCMV replication at the time of the study. The PI3K family is important for nearly all aspects of cell biology, acting at nearly all cellular membranes to regulate a wide range of signaling, membrane trafficking and metabolic processes (Jean and Kiger, 2014). The authors demonstrated that all 4 siRNAs from the siRNA pool against PI3K-C2A significantly reduced the number of pp28 positive cells; however, western blot analysis showed that knockdown of PI3K-C2A resulted in a modest decrease of pp28. Therefore, it was suggested that PI3K-C2A depletion is more likely to affect the production of viral or cellular factors important for productive replication rather than pp28 expression. Using electron microscopy, the authors also showed that knockdown of PI3K-C2A leads to an accumulation of capsids that had undergone secondary envelopment but was not released from the cell. Interestingly, both PI3K class I and class III have been shown to be involved in the intracellular signaling and secondary envelopment during HCMV infection, respectively, thus, the identification of PI3K class II as an important host factor for HCMV replication illustrates the involvement of all three classes of PI3K in HCMV replication and functional differences among classes of the same protein family (Johnson et al., 2001; Sharon-Friling and Shenk, 2014).

A virus expressing a fluorescence reporter can also be used as an indirect indication of infection and replication. For example, a GFP-expressing TB40/E virus (TB40/E-GFP) was developed by inserting an SV40 promoter-driven GFP cassette into the intragenic region between TRS1 and US34 (Umashankar et al., 2011). The use of a GFP-expressing virus can significantly reduce the time for the measurement of infection level, as long as the GFP expression represents or correlates with virus replication. Conventionally, siRNA screens were performed in one cycle of replication, where cells were transfected with siRNA, infected with virus and viral gene expression or viral genome copies were measured as an indication of virus replication. However, this approach can miss host factors that are involved in the later stages of the virus life cycle, such as virion assembly and egress. The approach of collecting supernatant following the first round of replication and assaying the supernatant for infectious virions on cells can address this issue. The aforementioned TB40/E-GFP virus was used in a medium-throughput siRNA screen, undertaken in our laboratory, using a library against 160 host genes involved in membrane organization (Lin et al., 2017; McCormick et al., 2018). The readout of GFP is a direct measure of infection but only an indirect measure of virus production. In contrast to conventional single-step screens, a two-step screening approach was used in this study. This involves the collection of supernatant at the end of the primary replication screen at 7 days post-infection (DPI), and the transfer of the supernatant to fresh untransfected cells, to measure production of infectious virus progeny by GFP expression. This approach independently measures virus replication and production using GFP readout which can be measured by plate cytometry. We showed a striking lack of correlation between the results from primary replication and virus production screens. For example, knockdown of some candidates reduced the level of GFP expression in primary replication but the level of infectious virus progeny was unaffected. Some of these factors may be involved in the regulation of GFP expression, instead of HCMV replication. Meanwhile, knockdown of other candidates had limited impact on primary replication, but virus production was substantially inhibited. These factors would represent false negative candidates in the conventional single-step screens. This analysis highlighted the advantages of the two-step screening approach in identifying host factors involved in both early (such as entry and early gene expression) and late (such as late gene expression and virion assembly/egress) stages of the virus life cycle. The screen successfully identified valosin containing protein, VCP, as an essential host factor for HCMV primary replication (Lin et al., 2017). VCP is a member of the hexameric AAA ATPase family and it plays a role in ubiquitin mediated signaling through remodeling target proteins, which are often degraded by proteasomes. It was found that VCP is involved in regulating expression of two viral major immediate-early genes, IE1 and IE2, and VCP knockdown resulted in loss of IE2 expression and subsequent loss of early and late gene expression. VCP was also shown to colocalised with the viral replication compartments in the nucleus and a small molecule inhibitor of VCP, NMS-873, was shown to be a potent HCMV antiviral. The same screen also identified novel host factors involved in virus assembly and egress, including ERC1, RAB4B, COPA, and COPB2 (McCormick et al., 2018). Knockdown of these candidates resulted in little or no reduction in primary replication but significantly reduced virus production. Further characterization studies showed distinct functions of these four host genes in the regulation of virus gene expression, DNA amplification, assembly and egress.

More recently we reported a high-throughput siRNA screen using a library targeting 6,881 host genes that are considered to be druggable and have high therapeutic potential (Lee et al., 2019). Using normal human dermal fibroblast cells (NHDF, similar to HFFs) and TB40/E-GFP, the screen identified 47 proviral and 68 antiviral factors involved in a broad range of cellular functions, such as transcription regulation by the mediator complex and proteasomal degradation. Asparagine synthetase (ASNS) was shown to be a previously unreported proviral factor for HCMV. It was found that knockdown of ASNS inhibited HCMV replication at an early stage and further analyses by western blotting showed that IE2 expression was inhibited in ASNS depleted cells. There are a number of studies demonstrating that amino acids function more than just in protein biosynthesis; instead, they are involved in a number of cellular pathways including cell cycle, apoptosis, and protein translation (Gong and Basilico, 1990; Kimball and Jefferson, 2006; Jewell et al., 2013; Meijer et al., 2015). The main function of ASNS is to produce the non-essential amino acid asparagine via glutamine and aspartate in an ATP-dependent manner. Asparagine has been shown to affect the cell cycle as a depletion of intracellular asparagine levels induces cell cycle arrest at the G1 phase (Gong and Basilico, 1990). However, since HCMV induces cell cycle arrest at the G1/S phase, the induction of cell cycle arrest by asparagine depletion does not explain an early inhibition of virus replication. Asparagine has also been shown to regulate uptake of amino acids in cancer cells and indirectly, as an amino acid exchange factor, regulates the mTOR pathway which controls protein synthesis (Krall et al., 2016). Previously, a series of studies showed that HCMV maintains mTOR activation during deprivation of essential amino acids through the binding and antagonizing of a major mTOR suppressor, tuberous sclerosis subunit complex 2 (TSC2), by the viral UL38 protein (Moorman et al., 2008; Terhune et al., 2010; Clippinger et al., 2011; Rodriguez-Sanchez et al., 2019). As with essential amino acid deprivation, mTOR activity, measured by the phosphorylation of S6 kinase, was also maintained by the virus following ASNS knockdown. Furthermore, knockdown of ASNS did not affect virus replication of another human herpesvirus, herpes simplex virus-1 (HSV-1), nor influenza A virus, suggesting ASNS depleted cells are still capable of continuing protein synthesis and supporting virus replication. These data suggest that there may be an unknown pathway regulated by intracellular asparagine levels impacting HCMV replication. To demonstrate that the effect of ASNS knockdown is due to decreased asparagine levels, we demonstrated that virus replication is fully rescued by adding exogenous asparagine at the time of infection in ASNS-depleted cells. Remarkably, upon ASNS depletion, virus replication can be completely rescued by supplying exogenous asparagine up to 7 days post-infection. This suggests that in ASNS depleted cells, HCMV is able to enter the cell and expresses IE1 but not IE2; thus, the virus remains dormant within the cell but retains the capacity for full replication days after initial infection and requires only asparagine to resume its replication.

There are studies in cancer cells showing the requirement of asparagine for cellular adaptation during glutamine starvation and for nucleotide biosynthesis (Zhang et al., 2014; Zhu et al., 2017). Furthermore, asparaginase, an enzyme that catalyzes asparagine into aspartate and ammonia, has been used to treat cancers including acute lymphoblastic leukemia, acute myeloid leukemia and non-Hodgkin's lymphoma (Hettmer et al., 2015; Bu et al., 2016; Egler et al., 2016). However, asparaginase resistance is reported in some cases of cancers and the use of ASNS inhibitors has been proposed and is currently in the development process (Gutierrez et al., 2006). Therefore, there is a possibility to repurpose anti-cancer drugs to treat HCMV based on the similar dependency upon asparagine between cancers and HCMV, or perhaps a simple dietary restriction in patients with high risk of HCMV infection may prevent the spread of the virus. The ability to “resume” virus replication by adding exogenous asparagine in ASNS-depleted cells also suggests the potential impact of asparagine levels and ASNS expression on the establishment and maintenance of HCMV latency. At this stage, it is unclear why HCMV requires asparagine to replicate efficiently whilst other herpesviruses such as HSV-1, does not.

Another way to measure virus infection in a high-throughput siRNA screen is to quantify levels of key viral transcripts by RNA-seq or reverse transcription-quantitative polymerase chain reaction (RT-qPCR) (Song et al., 2019). Recently, a high-throughput siRNA screen was reported which examined the role of cellular RNA-processing factors in immune regulation during HCMV lytic infection. Targeting 687 genes involved in cellular RNA processing, including RNA-binding proteins and ribonucleases using pooled siRNAs (4 siRNAs per gene), HFF cells were infected with HCMV low-passage strain Toledo and infection efficiency was quantified by measuring the mRNA levels of IE1 (immediate-early), UL44 (early), and UL99 (late) genes at 48 HPI (Song et al., 2019). Knockdown of 36 genes showed at least 3-fold decrease in mRNA levels of viral late gene UL99 expression. The authors followed up on one of the candidates, an RNA-binding protein Roquin encoded by the RC3H1 gene, and showed that the expression of Roquin is induced by HCMV and is required for efficient productive infection. Roquin plays important roles in both the innate and adaptive immune systems and promotes mRNA decay of transcripts with roles in immunity (Athanasopoulos et al., 2016). RNA-seq analysis performed in HCMV-infected and mock-infected Roquin depleted cells revealed increased production of pro-inflammatory cytokines such as interleukin-6 (IL-6) and IL-1B. However, the increased production of pro-inflammatory cytokines, chemokines, interferons (IFN) and interferon-stimulated genes (ISGs) was more drastic in infected cells, with cytokine levels being induced over 200-fold at 72 HPI. IFN and ISGs showed robust induction at 24 HPI in Roquin depleted cells. Cross-linking immunoprecipitation (CLIP)-seq analysis was performed to identify mRNA sequences targeted by Roquin during HCMV infection and found an enrichment of previously known Roquin targets such as pro-inflammatory cytokine IL-6 and chemokine CXCL2, as well as novel targets including dickkopf WNT signaling pathway inhibitor 1 (DKK1) and interferon regulatory factor 2 binding protein like (IRF2BPL). The mRNA targets identified in CLIP-seq analysis were also found enriched in Roquin depleted cells, suggesting that Roquin selectively targets these genes for downregulation during HCMV infection. Finally, in order to determine the specific Roquin targets involved in HCMV gene expression, 109 genes that showed increased expression upon Roquin depletion were selected for siRNA knockdown, along with Roquin depletion and infection monitored by quantifying the mRNA level of HCMV late gene UL146. Upon knockdown in HCMV-infected Roquin depleted cells, ANKRD52, CDK6, CFL2, and IRF1 showed a >50% rescue of UL146 gene expression. The authors suggested that Roquin may downregulate the expression of ANKRD52 to inhibit cellular miRNA-dependent regulation that may be involved in innate antiviral responses. Furthermore, suppression of CDK6 and CFL2 via Roquin can establish the cellular environment by controlling cell cycle and cytoskeleton, respectively. Since IRF1 is a well-characterized transcriptional activator of various cytokines, its role as a target by Roquin during HCMV replication was further studied. It was concluded that Roquin-mediated down-regulation of IRF1 is important for HCMV replication by mediating cytokine levels.

### shRNA Studies of HCMV

In addition to siRNA screens with individual gene knockdown in microwell plate formats, large-scale pooled shRNA expression lentiviral vector libraries provide another application for identification of host-virus interactions. shRNAs are about 80 nucleotides in length with a stem-loop structure similar to pre-miRNA (Fennell et al., 2014). They require processing by the Dicer protein before being incorporated into RISC. Unlike siRNAs which are usually transfected into cells as duplex RNAs, shRNAs are usually engineered into lentivirus vectors and integrated into genomic DNA for longer and more stable expression.

A high-throughput shRNA screen was employed to identify host factors involved in HCMV US11-mediated HLA class I degradation via the endoplasmic reticulum associated protein degradation (ERAD) pathway. A high-coverage shRNA library consisting of around 55,000 sequences (around 30 individual shRNAs per gene) that target all known human protein-encoding genes was generated (van de Weijer et al., 2014). Each shRNA lentivirus vector expresses mCherry. A U937 monocytic cell line co-expressing HCMV US11 and a chimeric HLA-A2 molecules with N-terminally tagged eGFP and Myc-epitope were used. Using the chimeric HLA-A2 molecules as an indication of US11-mediated degradation, the level of HLA class I molecule expression can be monitored by measuring GFP signal on the cell surface by flow cytometry or using a Myc specific antibody. Prior to shRNA transduction, it was confirmed that HCMV US11 expression significantly reduces the GFP expression from the chimeric HLA-A2 molecules. Following shRNA transduction, cells were sorted via GFP signal into GFP-bright and GFP-dim populations, and the GFP-bright population is further sorted for purity through selecting mCherry positive cells (cells transduced with an shRNA lentivirus vector) and GFP-bright cells. Around 2.8 million mCherry positive, GFP-bright cells per screen were selected for deep sequencing, along with 115 million mCherry positive, GFP-dim cells as control. GFP-bright populations are considered to have disrupted US11-mediated HLA class I downregulation. Two independent screens were performed and overlapping genes in the top 100 of two screens include coatomer subunits (e.g., COPA and COPB2) and proteasome subunits (e.g., PSMD1 and PSMC2). VCP was also identified as a clear hit, and this gene was identified in our medium-throughput siRNA screen (Lin et al., 2017). Several proteins involved in the ubiquitin pathway were identified in the GFP-bright populations but did not meet the strict cut-off requirements, including ubiquitin B, ubiquitin C, ubiquitin-activating enzyme E1 (UAE1) and the E2-conjugating enzyme UBE2J2. The authors focused on transmembrane protein 129 (TMEM129), and showed that TMEM129 is a novel E3 ubiquitin ligase and is important for HCMV US11-mediated HLA class I downregulation. The authors also demonstrated the involvement of UBE2J2, an E2 ubiquitin-conjugating enzyme, in the US11-mediated HLA class I downregulation, using shRNA and CRISPR/Cas9 techniques. A few years later, the same research groups further explored their study and performed a CRISPR/Cas9 screen to identify E2 ubiquitin-conjugating enzymes that are involved in the regulation of HCMV US2-mediated HLA class I downregulation. Thirty-six known human E2 enzymes were targeted, and 112 single guide RNAs (sgRNAs) were generated (van de Weijer et al., 2017). The same U937 monocytic cell line expressing an HLA-A2 molecule with an N-terminal eGFP tag was used to detect HLA class I expression mediated by HCMV US2. A similar screening strategy to the shRNA screen was employed, and UBE2G2 and UBE2D3 were identified as essential factors for US2-mediated HLA class I downregulation as sgRNAs targeting these two proteins led to an increased expression of GFP-tagged HLA-A2 molecules. However, sgRNAs targeting UBE2J2 resulted in further downregulation of HLA-A2 expression, suggesting that UBE2J2 counteracts the downregulation of HLA class I by US2 during HCMV infection. Interestingly, UBE2J2 was also identified in the shRNA screen, but it showed opposite functions for US11-mediated and US2-mediated HLA class I degradation. Unfortunately, the explanation on this was not given in the latter report; however, it is possible that UBE2J2 is involved in the regulation of HLA class I degradation between two viral proteins in an unknown mechanism.

### CRISPR/Cas9 Studies in HCMV

Cell-based RNAi has been the primary system for high-throughput gene perturbation, but some challenges and limitations persist, including toxicity and off-target effects (Mashalidis et al., 2013; Jadhav, 2014). siRNA off-target effects are common and can occur when unintended transcripts are silenced by siRNA through partial sequence complementarity or when passenger strands of the siRNA are loaded into RISC (Jackson and Linsley, 2010; Lam et al., 2015). siRNAs and/or their delivery vehicles can also trigger an inflammatory response through activation of Toll-like receptors (TLRs), causing an indirect inhibition of virus replication. However, pooling of multiple siRNAs to the same target transcript can help reduce off-target silencing, due to competition among the siRNAs in the pool (Kittler et al., 2007).

Unlike RNAi, CRISPR/Cas9 technology directly targets DNA for complete gene knockout and has been adapted in high-throughput screens as it overcomes some limitations of RNAi screens, including incomplete suppression of target genes and off-target effects (Pickar-Oliver and Gersbach, 2019). CRISPR/Cas9 allows specific manipulation of genomes based on the expression of modified Cas9 protein and single guide RNAs (sgRNAs). In nature, CRISPR is a prokaryotic immune response to invading DNAs such as bacteriophages and transposons. Specialized endonucleases called Cas proteins use CRISPR RNA (crRNA) to recognize and cleave target sequence. Depending on the Cas proteins, DNA can be nicked on one strand or cleaved on both strands. Cas9 protein was first found in *Streptococcus pyogenes* and reprogrammed for genome editing in mammalian cells (Pickar-Oliver and Gersbach, 2019). Containing two distinct nuclease domains, RuvC and HNH, Cas9 typically cleaves each strand with each nuclease domain, generating a DNA double-strand break (DSB) which is repaired by either homology directed repair (HDR) pathway or non-homologous end-joining (NHEJ) pathway. NHEJ repair is more common because the efficiency of HDR depends on the concentration of a homologous DNA template at the time of repair, the length of the homology, and the activity of the endogenous repair system (Lin et al., 2014; Maruyama et al., 2015). Due to the error-prone nature of the NHEJ repair pathway, mutations by insertion or deletion at the target site are often introduced, subsequently causing potential frameshift and downstream premature stop codons.

A human genome-wide CRISPR knockout (GeCKO) library has been used in a screen to identify host factors required for HCMV replication. The library targeted 19,050 human genes with over 120,000 sgRNA sequences (Wu et al., 2018). Two HCMV strains that only express viral trimer glycoprotein complex (gH/gL/gO), AD169 with a frame-shift insertion in UL131A and Merlin with a nonsense mutation in UL128, were used to infect HFFs. The surviving cells following infection were collected for sequence analysis of sgRNA enrichment, and sgRNAs targeting platelet-derived growth factor receptor alpha (PDGFRα) were enriched in cells infected with both trimer-only viruses. It was shown that the immunoglobulin-like domain 3 of PDGFRα, but not the kinase domain, is required for HCMV entry via trimeric complex. However, PDGFRα knockout fibroblast cells remain susceptible to HCMV that propagates in epithelial cells expressing pentameric complex (gH/gL/pUL128-pUL130-pUL131A).

A recent CRISPR/Cas9 screen identified an olfactory receptor family member, OR14I1, as an essential receptor for attachment, entry and infection of epithelial cells by pentameric complex expressing HCMV (Xiaofei et al., 2019). Using the same human GeCKO library (19,050 human genes, 6 sgRNAs per gene), two parallel screens were performed in either adult retinal pigment epithelial cell line 19 (ARPE-19) infected with epithelial-tropic TB40/E (expressing both trimeric gH/gL/gO and petameric gH/gL/pUL128-pUL130-pUL131A complexes) or human embryonic lung (HEL) fibroblast cells infected with fibroblast-tropic AD169 (expressing only trimeric gH/gL/gO complexes). Following CRISPR knockout, cells were repeatedly exposed to HCMV infection over 3 months at an MOI of 5. When 95% of respective cells had died, the surviving cells were expanded and subjected to a second round of infection. The surviving cells with sgRNA-induced resistance to HCMV were subjected to next-generation sequencing to identify enriched sgRNAs. sgRNAs against either OR14I1 or PDGFRα were found enriched in pentamaric complex expressing TB40/E infected cells. However, only sgRNAs against PDGFRα were enriched in trimeric complex expressing AD169 infected fibroblast cells, suggesting OR14I1 is not required for entry via trimeric complexes. OR14I1 is a G protein coupled receptor belonging to the olfactory receptor family, which is traditionally thought of as chemosensors for olfaction. However, it has been demonstrated previously that infectious agents exploit olfaction-related receptors for transmission and infection, such as murine CMV (MCMV) (Farrell et al., 2016). Therefore, it was intriguing that HCMV also requires an olfactory receptor for entry. Using a membrane flotation assay and immunoblots, it was shown that TB40/E, which expresses both trimeric and pentameric complexes, requires both OR14I1 and PDGFRα for optimal binding to epithelial cells. OR14I1 is predicted to have 4 peptide regions exposed on the cell surface and the first region, amino acid 1–26, was found to have the capability to interact with the pentameric complex, thereby limiting the binding and replication of TB40/E in epithelial cells. The authors also demonstrated that OR14I1 is required for the activation of adenylate cyclase (AC)/protein kinase A signaling pathway and the combination of OR14I1 binding and AC activity is required for TB40/E entry.

CRISPR/Cas9 offers a complete gene knockout by introducing mutations in target sites, instead of silencing gene expression by inducing mRNA degradation or disrupting translation. Loss-of-function CRISPR/Cas9 screens can be performed using sgRNAs against exons, non-coding regions or transcription factor motifs (Pickar-Oliver and Gersbach, 2019). There are several factors that can affect the efficacy of the CRISPR/Cas9 screen, including off-target cutting, incidence and efficiency of HDR vs. NHEJ, and Cas9 activity (Lino et al., 2018). However, one of the major challenges of the two screens discussed in this review is bias toward the enrichment of sgRNAs targeting surface receptors for virus entry. Although the importance of PDGFRα for trimeric complex expressing HCMV entry was successfully validated and OR14I1 as a novel receptor for pentameric complex expressing HCMV was identified, the enrichment of sgRNAs against other cellular factors that may be involved in viral gene expression or other processes is less substantial. There are potential approaches to circumvent the bias of currently published CRISPR. For example, instead of using pooled lentiviral vectors and survival assays, high-throughput microplate systems, similar to siRNA screens, with each well containing sgRNAs targeting the same gene can be used. Furthermore, fluorescent activated cell sorting (FACS) can also be adapted to measure virus infection and sort cells based on the level of virus replication prior to sequencing for sgRNA enrichment. There is also a future revenue in single cell sequencing following a CRISPR screen as it helps to dissect cellular differences at higher resolution and understanding how individual cells respond to infection. Adaptation of other high-throughput techniques such as liquid handling automation for liquid dispensation and collection in microplates and high-content microscopy and improvement on design of sgRNA libraries may lead to a new generation of CRISPR/Cas9 screens with higher rates of hit identification.

### miRNA Studies in HCMV

A more indirect, but potentially powerful approach for the identification of novel host-virus interactions is through miRNA target discovery. Herpesviruses express their own viral miRNAs that can target both viral and cellular transcripts. Viruses also modulate the expression of cellular miRNAs, thereby indirectly affecting host gene expression. By identifying targets of viral miRNAs, novel host factors important for virus replication can be discovered; in essence, using the virus to indicating which host genes are important for its replication cycle.

miRNAs are initially transcribed as long primary miRNA (pri-miRNA) with one or more sequences that can hybridize and form hairpin structures (Fennell et al., 2014). The pri-miRNA is processed by RNase Drosha/Pasha in the nucleus to produce pre-miRNAs which have individual stem-loop hairpin structures. Pre-miRNAs are exported to the cytoplasm where they are processed by the Dicer protein to form short double-stranded siRNA-like miRNA duplexes, which are 19–25 nucleotide long (Tomari and Zamore, 2005; Lam et al., 2015). A single-stranded mature miRNA is then incorporated into the RNA-induced silencing complex (RISC) which uses the miRNA sequence as a guide to recognize complementary mRNA transcripts and cleaves the mRNA via Argonaute protein which is within RISC. miRNAs modulate gene expression primarily through targeting the 3′ untranslated region (3′UTR) of genes, and often have multiple targets through partial complementarity (Fennell et al., 2014).

Previously, our lab performed a systematic RISC immunoprecipitation (RISC-IP) analysis to identify host factors targeted by HCMV-encoded miRNAs (Pavelin et al., 2013). By pulling down RISC using an argonaute 2 (AGO2) specific antibody and analyzing mRNA transcripts by microarray, ATP6V0C, a vacuolar ATPase complex involved in acidification of endosomal compartments, was identified as a novel target of viral mir-US25-1. Interestingly, further characterization studies using siRNA against ATP6V0C showed that depletion of ATP6V0C resulted in almost complete loss of infectious virus production, suggesting that HCMV targets a crucial host factor required for productive virus replication. ATP6V0C was also found to be required for the formation of virion assembly compartment (VAC) during HCMV infection (Pavelin et al., 2017). Currently, it is still not completely understood why HCMV targets ATP6V0C. We suggested that it may be related to the establishment of latency or immune evasion as endosomal acidification is required for efficient MHC class II antigen presentation, but a mechanism remains to be established.

Subsequently, argonaute-crosslinking and immunoprecipitation followed by high-throughput screening (AGO-CLIP-seq, also known as Ago HITS-CLIP) was performed in HCMV strain Towne_long_ infected HFFs in order to identify miRNA-target interaction sites (Kim et al., 2015). Compared to the RISC-IP analysis, the AGO-CLIP-seq involves UV-crosslinking of RNA with argonaute (AGO) proteins and sequencing of mRNAs and small RNAs. Furthermore, a pan-AGO-specific monoclonal antibody was used instead of an AGO2-specific antibody; thus, the results will reveal miRNAs associated with all AGO proteins rather than specific interactions with AGO2. In mammals, there are four AGO proteins but only AGO2 is catalytically active and can degrade target transcripts (Meister, 2013). The AGO-CLIP-seq identified 3,909 human targets of the viral miRNAs (HTVs) from data collected at 24, 48, and 72 HPI, and these target gene groups contain the peak clusters with enrichment higher in infected samples than in uninfected samples. Thirty high-confidence candidates were selected for validation by quantitative real-time PCR (qRT-PCR) to examine the fold reduction in mRNA expression following transfection with targeting viral miRNAs into HFFs. At 48 h post-transfection, 26 of 30 were significantly downregulated. mRNA levels of most of these targets were recovered or noticeably higher when infected with miRNA-mutant viruses. The authors focused on two pathways that are targeted by viral miRNAs: cell cycle/apoptosis/autophagy and interferon JAK/STAT signaling, and validated their results using viral miRNA mimics and luciferase reporter assays for the 3′UTR of target mRNAs. Finally, the targetomes of human miRNAs were also investigated using the same dataset and it was found that increased expression of human miRNAs usually leads to decreased overall mRNA expression of targets; however, decreased expression of human miRNAs does not increase the overall mRNA expression. These experiments help dissect complex miRNA-mediated host manipulation during HCMV lytic infection.

## Concluding Remarks and Future Directions

**Figure 1:**
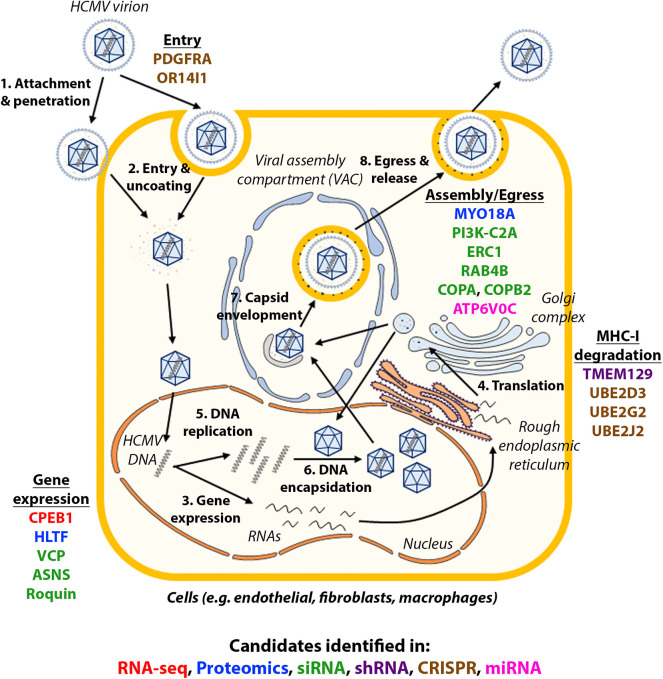
Host factors identified in high-throughput studies and their role in HCMV infection. The life cycle of HCMV is summarized in the figure and candidates identified in each type of high-throughput studies were shown in their approximate functional location. Candidates identified in each type of high-throughput studies are in: red (RNA-seq), blue (proteomics), green (siRNA), purple (shRNA), brown (CRISPR), pink (miRNA).

Systems virology, involving high-throughput molecular profiling and bioinformatics, has highlighted the complexity of host-virus interactions and contributed to our understanding of how HCMV works, novel cellular functions and potential new drug targets (Law et al., 2013). The screens discussed in this review are summarized in Table 1. The validated candidates in each screen are illustrated in Figure 1, with each color showing the type of screens the candidate was identified in.

**Table 1:** Summary of high-throughput studies.

	**References**	**Experimental techniques^+^**	Cell type^*^	**HCMV strains for screens**	**Detection method of infection**	**Number of “hits”**
RNA-seq	Batra et al. (2016)	Affymetrix microarrays; poly-A selected RNA-seq; TAIL-seq	NPCs, HFFs, Ecs	TB40/E and Towne	HCMV mRNA expression pattern	Over 2,000 altered host RNA splicing events in infected HFFs and NPCs
Proteomics	Weekes et al. (2014)	TMT proteomics of 1,184 surface proteins and 7,491 cellular proteins	HFFFs (HFFs)	Merlin	HCMV protein expression profile	Not specified
	Jean Beltran et al. (2016)	Subcellular fractionation, TMT-based and label-free mass spectrometry and confocal microscopy	HFFs	AD169-GFP	GFP expression from the virus	796 proteins downregulated and 593 upregulated up to 96 HPI; 374 proteins showed translocation upon infection
	Arend et al. (2017)	Multiplexed kinase inhibitor bead-mass spectrometry (MIB-MS) quantitatively measure perturbations in 244 cellular kinases	MRC-5	AD169 and TB40/E	Infection not measured	53 kinases upregulated and 51 downregulated at multiple timepoints post-infection
	Nightingale et al. (2018)	TMT proteomics with proteasomal (MG132) and lysosomal (leupeptin) inhibitors; SILAC; RNA-seq	Immortalized HFFs (HFFF-TERTs)	Merlin	HCMV protein expression profile	131 proteins were rescued by MG132 and 28 proteins by leupeptin within 24 h of infection
	Jean Beltran et al. (2018)	Targeted mass spectrometry against peroxisome proteins	HFFs	AD169	Viral protein expression profile of IE1, pUL26 and pp65	52 peroxisome proteins showed increased expression from 24 HPI
siRNA screens	Polachek et al. (2016)	An siRNA library, in a pool of 4 siRNAs, targeting 789 human kinases and phosphatases	HFFs	AD169	Immunoflurescencely labeling pp28	26 proviral and 25 antiviral hits
	Lin et al. (2017)	An siRNA library, in a pool of 4 siRNAs, targeting 140 membrane trafficking genes and 20 other selected genes	NHDF (HFFs)	TB40/E-GFP	SV40 promoter driven GFP expression from the virus	Not specified
	McCormick et al. (2018)	An siRNA library, in a pool of 4 siRNAs, targeting 156 host genes	NHDF (HFFs)	TB40/E-GFP	SV40 promoter driven GFP expression from the virus	11 proviral and 31 antiviral hits from the virus production screen
	Lee et al. (2019)	An siRNA library, in a pool of 4 siRNAs, targeting 6,881 host genes with high therapeutic potential	NHDF (HFFs)	TB40/E-GFP	SV40 promoter driven GFP expression from the virus	47 proviral and 68 antiviral hits
	Song et al. (2019)	An siRNA library, in a pool of 4 siRNAs, targeting 687 host genes involved in cellular RNA processing	HFFs	Toledo	RT-qPCR of IE1, UL44, UL99 at 48 HPI	36 proviral genes based on UL99 expression
shRNA screens	van de Weijer et al. (2014)	A high-coverage shRNA library of around 55,000 sequences (30 shRNAs per gene)	U937	N/A; Cells expressing HCMV US11 proteins	Cells engineered to express chimeric HLA-A2 molecules with eGFP and Myc tags; cell sorting by FACS	Not specified; top 100 enriched sgRNAs/genes from both screens shown
CRISPR screens	van de Weijer et al. (2017)	A CRISPR library of 112 sgRNAs targeting 36 known human E2 enzymes	U937	N/A; Cells expressing HCMV US2 proteins	Cells engineered to express eGFP-HLA-A2 molecules; cell sorting by FACS	Knockout of 2 genes showed rescue expression of eGFP-HLA-A2 and knockout of 1 gene showed further downregulation of eGFP-HLA-A2
	Wu et al. (2018)	A human genome-wide GeCKO library targeting 19,050 human genes with over 120,000 sgRNAs	HFFs	AD169 with UL131A insertion and Merlin with UL128 nonsense mutation	Sequence analysis of sgRNA enrichment in surviving cells	Not specified; top 10 enriched sgRNAs shown
	Xiaofei et al. (2019)	A human genome-wide GeCKO library targeting 19,050 human genes with over 120,000 sgRNAs	ARPE-19, HEL	TB40/E and AD169	Sequence analysis of sgRNA enrichment in surviving cells	312 candidates (≥3 sgRNAs, each with ≥20 reads) in ARPE-19 screen
miRNA screens	Pavelin et al. (2013)	RISC-IP using an AGO2 specific antibody	NHDF (HFFs)	AD169 and TR	N/A	686 transcripts were enriched over 2-fold in AD169-infected cells; 442 transcripts enriched in TR-infected cells
	Kim et al. (2015)	AGO-CLIP-seq using a pan-AGO antibody	HFFs	Towne_LONG_	N/A	3,909 HTVs^**^ from data collected at 24, 48, and 72 HPI

+ *TMT, tandem mass tag; RISC-IP, RNA-induced silencing complex immunoprecipitation*.

* *NPCs, neural progenitor cells; HFFs, human foreskin fibroblasts; ECs, endothelial cells; HFFFs, human fetal foreskin fibroblast; HFFF-TERTs, HFFF immortalized with human telomerase; NHDF, normal human dermal fibroblast; HEL, human embryonic lung fibroblast*.

** *HTVs, human targets of the viral miRNAs*.

*N/A, not applicable*.

With advances in sequencing, mass spectrometry and genome editing, high-throughput studies become more efficient and accurate in the identification of host-virus interactions. Despite the discovery of novel host factors important for virus replication, the precise functions and roles of these factors remain to be fully elucidated. The potential of these factors as antiviral targets will also require extensive examinations *in vivo* prior to proceeding to clinical trials. The development of host-oriented antivirals will have lower rates of the emergence of drug-resistant virus, thereby providing a better option for therapeutics. However, drug toxicity in the host is a major challenge most novel antivirals need to overcome because some host factors are required for normal cellular functions.

Integrating the various high throughput approaches, from RNA-seq to proteomics to phenotypic screens, represents an attractive future goal of systems approaches in HCMV research as well as systems research in general. With improving pathway analysis, integrated systems approaches may lead to major advances in our understanding of basic virus biology, cell biology and the discovery of novel antiviral therapies.

## Author Contributions

C-HL wrote the manuscript. All authors contributed to manuscript revision, read, and approved the submitted version.

## Conflict of Interest

The authors declare that the research was conducted in the absence of any commercial or financial relationships that could be construed as a potential conflict of interest.

## Abbreviations

3′UTR: 3′ untranslated regions
AGO2: Argonaute 2
AGO-CLIP-seq: Argonaute-crosslinking and immunoprecipitation sequencing
AMPK: 5′ AMP-activated protein kinase
ANAPC1: Anaphase-promoting complex subunit 1
ANKRD52: Ankyrin repeat domain 52
APA: Alternative cleavage and polyadenylation
ARPE-19: Adult retinal pigment epithelial cell line 19
AS: Alternative splicing
ASNS: Asparagine synthetase
ATP6V0C: ATPase H+ transporting V0 Subunit C
BRD2: Bromodomain-containing protein 2
CD4: Cluster of differentiation 4
CDK6: Cyclin dependent kinase 6
CFL2: Cofilin 2
CHEK1: Checkpoint kinase 1
CLIP-seq: Cross-linking immunoprecipitation-sequencing
COPA: Coatomer protein complex subunit alpha
COPB2: Coatomer protein complex subunit beta 2
CPEB1: Cytoplasmic polyadenylation element binding protein 1
CRISPR: Clustered regularly interspaced short palindromic repeats
crRNA: CRISPR RNA
CUL4: Cullin 4
CXCL2: Chemokine (C-X-C motif) ligand 2
DKK1: Dickkopf WNT signaling pathway inhibitor 1
DPI: Days post-infection
DSB: Double-strand break
ECs: Endothelial cells
eGFP: Enhanced green fluorescent protein
ERAD: Endoplasmic reticulum associated protein degradation
ERC1: ELKS/RAB6-interacting/CAST family member 1
ERK/MAPK: Extracellular signal-regulated kinase/mitogen-activated protein kinase
EXOSC10: Exosome component 10
FACS: Fluorescent activated cell sorting
GeCKO: Genome-wide CRISPR knockout
HCMV: Human cytomegalovirus
HDR: Homology directed repair
HEL: Human embryonic lung fibroblast cells
HFFs: Human foreskin fibroblast cells
HLTF: Helicase-like transcription factor
HPI: Hours post-infection
HSV-1: Herpes simplex virus 1
HTVs: Human targets of the viral miRNAs
IFN-: Interferon-
IL-: Interleukin-
IRF1: Interferon regulatory factor 1
IRF2BPL: Interferon regulatory factor 2 binding protein like
IRF9: Interferon regulatory factor 9
ISGs: Interferon stimulated genes
Jak1: Janus kinase 1
kb: Kilobases
mDCs: Myeloid dendritic cells
MELK: Maternal embryonic leucine zipper kinase
miRNA: MicroRNA
mRNA: Messenger RNA
mTOR: Mammalian target of rapamycin
MYO18A: Myosin XVIIIA
NEDD4: Neural precursor cell expressed, developmentally downregulated protein 4
NGS: Next generation sequencing
NHDF: Normal human dermal fibroblast cells
NHEJ: Non-homologous end-joining
NPCs: Neural progenitor cells
OR14I1: Olfactory receptor family 14 subfamily I member 1
PDGFRα: Platelet-derived growth factor receptor alpha
PHLDA1: Pleckstrin homology like domain family A member 1
PI3K-C2A: Phosphatidylinositol 3-kinase, Class 2, Alpha
PI4K2B: Phosphatidylinositol 4-kinase type 2-beta
PIK3C3: Phosphatidylinositol 3-kinase catalytic subunit type 3
pri-miRNA: Primary miRNA
pSILAC: Pulsed stable isotope labeling with amino acids in cell culture
PSMC2: Proteasome 26S subunit, ATPase 2
PSMD1: Proteasome 26S subunit, non-ATPase 1
RAB4B: Ras-related GTP-binding protein 4B
RISC: RNA-induced silencing complex
RISC-IP: RNA-induced silencing complex-immunoprecipitation
RNAi: RNA interference
RNA-seq: RNA-sequencing
rRNA: Ribosomal RNA
RT-qPCR: Reverse transcription-quantitative polymerase chain reaction
sgRNA: Single-guide RNA
shRNA: Short hairpin RNA
siRNA: Small interfering RNA
STAT2: Signal transducer and activator of transcription 2
TGFBR1: Transforming growth factor beta receptor 1
TLRs: Toll-like receptors
TMEM129: Transmembrane protein 129
TMT: Tandem mass tags
TSC2: Tuberous sclerosis subunit complex 2
TXNIP: Thioredoxin interacting protein
UAE1: Ubiquitin-activating enzyme E1
UBE2J2: Ubiquitin conjugating enzyme E2 J2
VAC: Virion assembly compartment
VCP: Valosin containing protein
WNK4: WNK lysine deficient protein kinase 4.
